# Clinical Data on Canabinoids: Translational Research in the Treatment of Autism Spectrum Disorders

**DOI:** 10.3390/biomedicines10040796

**Published:** 2022-03-29

**Authors:** Laura D. Carreira, Francisca C. Matias, Maria G. Campos

**Affiliations:** 1 Observatory of Drug-Herb Interactions, Faculty of Pharmacy, Health Sciences Campus, University of Coimbra, Azinhaga de Santa Comba, 3000-548 Coimbra, Portugal; lauracarreira98@gmail.com; 2 Coimbra Institute for Biomedical Imaging and Translational Research, Institute of Nuclear Sciences Applied to Health (ICNAS), Health Sciences Campus, University of Coimbra, Azinhaga de Santa Comba, 3000-548 Coimbra, Portugal; franciscacamposmatias@gmail.com; 3 Coimbra Chemistry Centre (CQC, FCT Unit 313) (FCTUC), University of Coimbra, Rua Larga, 3004-535 Coimbra, Portugal

**Keywords:** cannabinoids, cannabidiol, clinical trial, anxiety, FAAH, social interaction, inflammation, gamma-aminobutyric acid, glutamate, neuroimaging

## Abstract

Translational research made with *Cannabis sativa* L. and its biocompounds provides data for some targeted diseases, as also symptoms associated with Autism Spectrum Disorders (ASDs). The main compounds ∆9-tetrahydrocannabinol (THC) and cannabidiol (CBD), are capable of modulating the endocannabinoid system since its dysregulation interferes with the pathophysiology of ASDs there are clinical evidence for its potential use in the treatment of the disease. Conventional therapy still has limitations, as it does not always treat the central symptoms, and there are many patients who do not respond to treatment, which demands more research on new therapies. Through the analysis of published literature on this topic, it is verified that cannabinoids, in particular CBD, improves symptoms associated with common comorbidities in ASDs. Some studies also demonstrate the therapeutic potential of these compounds in the treatment of central symptoms of autism. In addition, cannabinoid therapy to ASDs is associated with low adverse effects and a reduction in concomitant medication. Although it appears to be promising, it is essential to do the translation of this data into clinical research and some of its potential and critical gaps are discussed in this review pointing to large-scale and long-term clinical trials that should include more patients and homogeneous samples.

## 1. Introduction

Up to date, the translational research applied to humans made with *Cannabis sativa* L. biocompounds gave a proof of concept in a new approach for some, already signalized diseases as, for instance, Autism Spectrum Disorders (ASDs). The plant is endemic from Asia [1] (p. 412) and has been used for thousands of years for various purposes ([1,2] (p. 412), (p. 234)). In the medicinal context, it is considered that the drug consists of female flowers of the plant where there is a high amount of glandular trichomes that contain pharmacologically active compounds [3] (p. 300). Among these compounds stand out the phytocannabinoids, biosynthesized by *C. sativa* L. [3] (p. 305), that bind to cannabinoid receptors modulating the endocannabinoid system [1] (p. 414). The most studied are cannabidiol (CBD) and ∆9-tetrahydrocannabinol (THC) [1] (p. 413), which have been attributed several therapeutic properties, including the reduction of symptoms associated with ASDs [4] (pp. 6–11).

ASDs are a group of complex neurodevelopmental diseases characterized by persistent deficits in communication and social interaction, as well as restricted and repetitive patterns of behavior, interests, and activities [5] (pp. 50–59). In addition to these central symptoms, ASDs are often accompanied by comorbidities that have a high impact on the quality of life of patients and their caregivers [6] (p. 8). Currently, there are no approved drugs for the treatment of the central symptoms of ASDs and [7] (p. 2), therefore, conventional therapy involves the use of drugs that can mitigate some of the associated symptoms, as well as behavioral and educational therapies. Due to the high number of refractory patients and the incidence of serious adverse effects [7] (p. 1), treatments that improve or replace conventional ASD therapy have been increasingly sought [8,9] (pp. 100–101) (p. 3). Research in this area suggests that the use of phytocannabinoids, in particular, CBD, could become a possible therapeutic strategy in these situations [4] (pp. 10–11).

Thus, the main objective of the present work is to synthesize and analyze the existing literature to date mainly associated with Clinical trials evidence, regarding the role of the endocannabinoid system in ASDs, as well as the therapeutic potential of cannabinoids in treatment.

## 2. Materials and Methods

A non-systematic literature search on MEDLINE (http://www.ncbi.nlm.nih.gov/pubmed) was conducted at 30 June 2021 by using the following subject headings/keywords or MeSH terms where available: (a) ‘cannabis’ OR ‘cannabinoids OR ‘phytocannabinoids’ OR ‘cannabidiol’ OR ‘CBD’ OR ‘THC’ and (b) ‘autism’ OR ‘autism spectrum disorder’ OR ‘ASD’.

The authors screened all retrieved papers and included all original studies written in English, published as full papers or abstracts, and met the selection criteria. The selection criteria included studies with participants with a diagnosis of ASD treated with *C. sativa* L. or cannabinoids, such as CBD, CBDV, THC, etc., with or without a comparison group. Due to scarce data and since the reported outcomes were expected to vary, no specific outcomes were defined to facilitate a comprehensive evaluation of the available studies in this area. All potentially eligible studies were considered regardless of study design. At initial screening, the studies were assessed independently for potential inclusion by title and abstract. Following the initial screening, the full text of eligible publications was examined, and a final decision for inclusion was made. In addition, citations in the selected articles were reviewed by authors for identifying additional eligible articles. The articles of preclinical studies and reviews were excluded. The authors extracted information about studies design, characteristics of participants, characteristics of the treatment, and observed outcomes and adverse effects. The authors also report data regarding ongoing studies, as retrieved in ClinicalTrials.gov. The results of the study were presented in a narrative summary and summarized in tables organized around the characteristics of the studies.

## 3. Results

Brief information about the plant and its bioactive compounds, the description of the cannabinoid system, including its receptor, and the biomolecules involved in all these mechanisms is provided below for a better understanding of the implications that they have in Clinical Trials. Likewise, the description of the ASDs main symptomatology, etiology, and treatments. We will use this information to discuss the gaps in data available as well as propose further studies that should be done in order to assure the safe use of this therapeutic in ASDs.

### 3.1. Cannabis, Flos

To date, a monograph of the drug “Cannabis, flos” is not available in the European Pharmacopoeia but it is largely followed by the one in German Pharmacopoeia, where it is defined as “dried shoot apices of female *Cannabis sativa* L. (Cannabaceae) plants. The drug contains not less than 90.0 and not more than 110.0 percent of cannabinoid quantities indicated in the label, such as ∆9-tetrahydrocannabinol and cannabidiol, as well as cannabinoid-carbon acids, such as ∆9-tetrahydrocannabinolic acid and cannabidiolic acid, calculated as ∆9-tetrahydrocannabinol (C_21_H_30_O_2_; Mr 314.5) and cannabidiol (C_21_H_30_O_2_; Mr 314.5), referenced to dried drug” [10].

**Figure 1:**
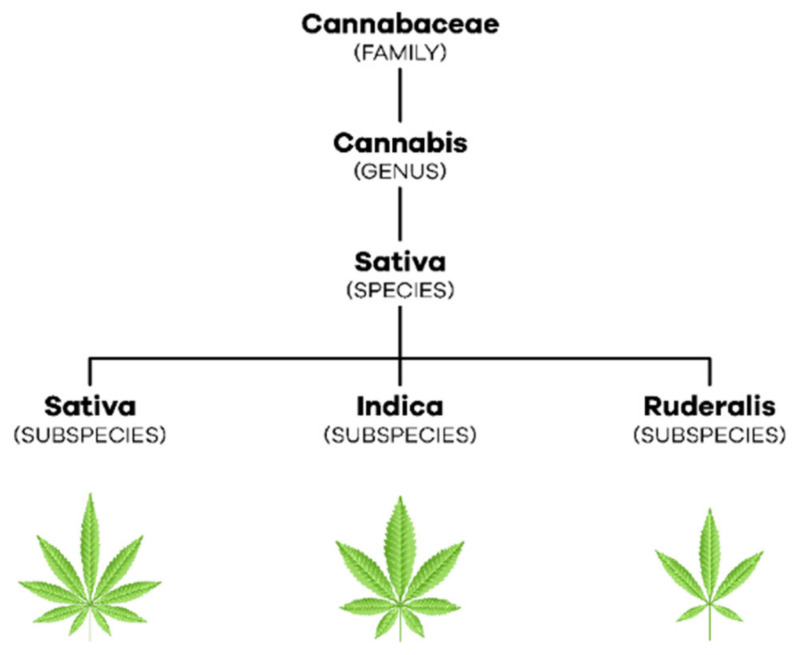
Taxonomic organization of *C. sativa* L.

Cannabis is a dioecious annual plant [3] (p. 304) that belongs to the Cannabaceae family, which includes about 170 species [11] (pp. 203–204). With regard to taxonomy, in 1753, Linnaeus began by describing only one species of this plant, Cannabis sativa. Later, in 1785, Lamarck considered the existence of two species of cannabis, Cannabis sativa, which came from the West, and *Cannabis indica*, which came from India, Southeast Asia, and Southern Africa ([2,11] (p. 236), (pp. 207–208)). Only in the 19th century emerged the reference to a new species of cannabis, *Cannabis ruderalis* [2] (p. 236). Currently, most authors consider that there is only one species, *C. sativa* L., which comprises three subspecies, *C. sativa* subsp. sativa, *C. sativa* subsp. *indica* and *C. sativa* subsp. *ruderalis* [12] (p. 3) (Figure 1). *C. sativa* subsp. *sativa* is a tall, branched plant that can reach more than five meters in height, it produces a large amount of THC relative to CBD and its terpenoid profile gives it a sweet smell. Furthermore, *C. sativa* subsp. *indica*, which has wider leaves, only reaches one to two meters in height, produces a similar amount of THC and CBD and its terpenoid profile causes it to have a bitter odor. *C. sativa* subsp. *ruderalis* is the smallest plant reaching only one meter in height and producing the least amount of cannabinoids ([11,13] (p. 208), (p. S2)).

The drug consists of the flowers of *C. sativa* L. female plants, because they have more glandular trichomes that accumulate in their interior secondary metabolites responsible for the studied bioactivities, namely phytocannabinoids and terpenoids ([1,3] (p. 413), (p. 304)). The content of these compounds is influenced by environmental conditions like temperature, humidity, presence of pests, and soil composition. Terpenoids are responsible for the characteristic smell of the plant, while phytocannabinoids are defense agents and responsible for the interaction with other species, whether animal or plant [3] (p. 305). However, for medicinal use, the plant’s growing conditions comply with specific guidelines covered by the current legislation in order to produce plants always with the same characteristics and totally free of contaminants, from the soil and from the environment [14] (pp. 1–11).

### 3.2. Endocannabinoid System

The endocannabinoid system is involved in the organism’s homeostasis [1] (p.413) through the modulation of multiple organs comprising the cardiovascular (CVS), central nervous (CNS), peripheral nervous (PNS), endocrine, reproductive, immune and digestive systems ([15,16] (p. 2) (p. 554)).

#### 3.2.1. Cannabinoid Receptors

The two main receptors of the endocannabinoid system are the cannabinoid receptor type 1 (CB1), encoded by the CNR1 gene which is made with 472 amino acids, and the cannabinoid receptor type 2 (CB2), encoded by the CNR2 gel and composed of 360 amino acids [17] (p. 2). Both are G protein-coupled metabotropic receptors ([18,19] (p. 2), (p. 3)) and their expression varies depending on the body region [16] (p. 559).

The CB1 receptor is found mainly in CNS neurons with high expression density in the basal ganglia, cerebral cortex, hippocampus, and cerebellum [17] (p. 5). Thus, it is involved in motor control, cognitive functions, movement coordination, learning, and memory. It is also expressed in PNS and peripheral tissues, namely, in sympathetic nerve terminals, gastrointestinal tract, lungs, musculoskeletal tissue, reproductive and immune system, among others [16] (p. 559). Furthermore, its hepatic and cardiovascular expression increases in the presence of pathological conditions [17] (p. 5).

In turn, the CB2 receptor is mostly found in peripheral cells of the immune system [20] (p. 1140) such as the spleen, tonsils, and thymus [16] (p. 559). It is also expressed in lower concentrations in the pancreas, liver, bone marrow, bones, and skin [21] (p. 3). At the cerebral level, it is found in low amounts, however, in inflammatory states, its expression increases both in microglia and in other glial cells [3] (p. 309).

#### 3.2.2. Endocannabinoids

The most studied endocannabinoids are *N*-arachidonylethanolamine or anandamide (AEA) and 2-arachidonylglycerol (2-AG) [3] (p. 308). AEA is known to be a high-affinity partial agonist for the CB1 receptor and with very low affinity for the CB2 receptor, while 2-AG is a full agonist with low to moderate affinity for the two cannabinoid receptors [17] (p. 2).

**Figure 2:**
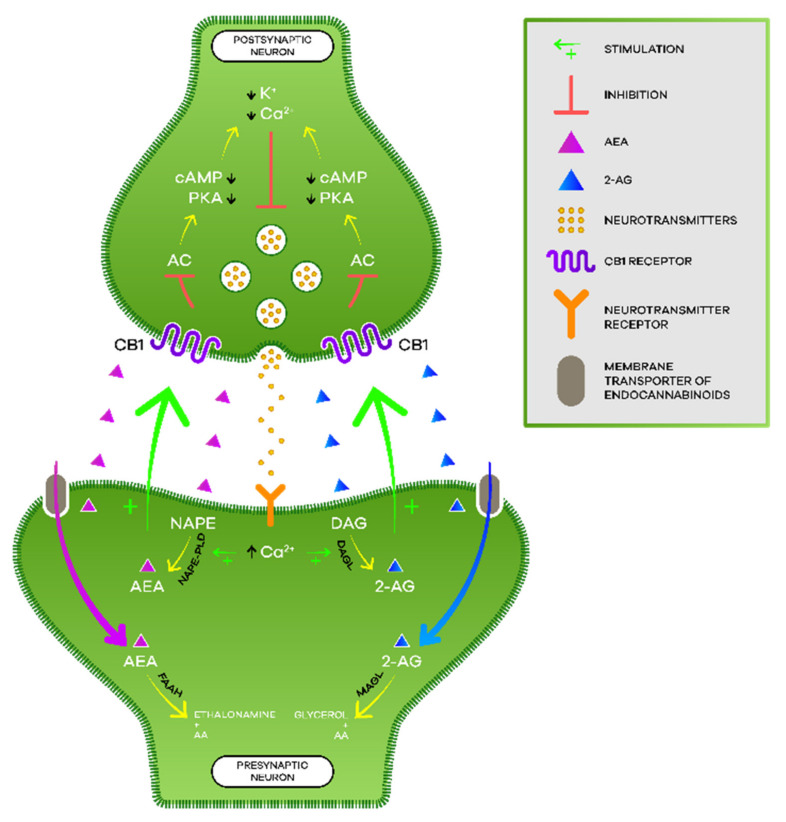
Endocannabinoid signaling.

Neurotransmission in the postsynaptic neuron leads to an increase in intracellular calcium and, consequently, to the production of endocannabinoids from their lipid precursors [22] (p. 4). AEA is produced from *N*-acyl-phosphatidylethanolamine (NAPE) by *N*-acyl-phosphatidylethanolamine-specific phospholipase D (NAPE-PLD) while 2-AG is formed from diacylglycerol (DAG) by diacylglycerol lipase (DAGL) [19] (p. 3). As lipid molecules, they are able to cross the plasma membrane and travel in a retrograde direction to the presynaptic terminals [17] (p. 4). There, the endocannabinoids bind and activate cannabinoid receptors, which, in turn, inhibit the adenylate cyclase (AC) enzyme, decreasing the formation of cyclic adenosine monophosphate (cAMP) and the activation of protein kinase A (PKA) [18] (pp. 2–3). Thus, hyperpolarization occurs by opening potassium channels, the consequent closure of calcium channels, and the release of neurotransmitters ceases [22] (p. 4). The endocannabinoids get retaken by the membrane transporter of endocannabinoids and then hydrolyzed by the respective enzymes [23] (pp. 2–3). AEA is catabolized by fatty acid amide hydrolase (FAAH), giving rise to AA and ethanolamine while 2-AG is catabolized by monoacylglycerol lipase (MAGL) giving rise to AA and glycerol ([3,22] (p. 308), (p. 4)) (Figure 2).

Moreover, the activation of cannabinoid receptors increases the activity of mitogen-activated protein kinase (MAPK) associated with the control of synaptic plasticity, cell migration, and neuronal growth. Additionally, within intracellular compartments, we find cannabinoid receptors, such as in mitochondria, which, when activated, are capable of modifying levels of reactive oxygen species, calcium, and adenosine triphosphate (ATP) [22] (p. 4).

#### 3.2.3. Phytocannabinoids

**Figure 3:**
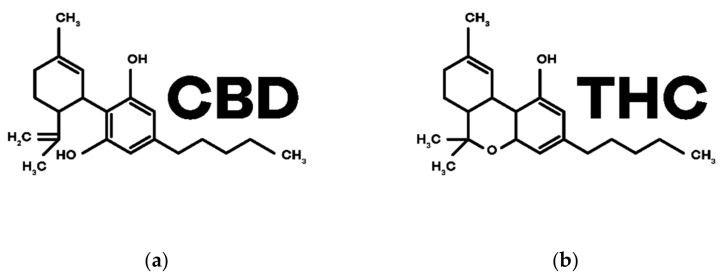
Chemical structures of the main phytocannabinoids in *Cannabis sativa* L.: (**a**) CBD; (**b**) THC.

Phytocannabinoids are biosynthesized in *C. sativa* L. and, currently, there are more than 100 phytocannabinoids identified among the more than 500 compounds discovered in this plant. Its distribution is variable but phytocannabinoids accumulate, in large quantities, in the trichomes present in female flowers as previously referred to in this text [3] (p. 305). Among them, THC and CBD are the most studied ones. The structures are in Figure 3.

CBD was the first phytocannabinoid to be isolated in its pure form, in 1899, by Thomas Hill Easterfild. [24] (p. 922). Even though it does not have psychoactive properties, it has shown potential for various therapeutic effects and has been extensively investigated for the treatment of various pathologies [25] (pp. 8–13). It is a negative allosteric modulator of CB1 and CB2 receptors and has the ability to inhibit the reuptake and degradation of the endocannabinoid AEA ([3,4] (p. 306), (p. 6)). In addition, CBD interacts with other non-cannabinoid receptors such as serotonin (5-HT) receptors, orphan G protein-coupled receptors (GPCRs), adenosine A1 receptors, nuclear receptors activated by peroxisome proliferators type γ (PPARγs), transient receptors potential cation channel subfamily V member 1 (TRPV1s) and also α1 and α3 glycine receptors ([26,27] (p. 4), (p. 1)).

THC, in turn, is the main component of the female flowers of *C. sativa* L. [28] (p. 1), being a partial agonist of the receptors CB1 (responsible for the psychoactive effects) and CB2 intervening in the immunological and anti-inflammatory effects [3] (p. 307).

### 3.3. Physiological and Therapeutic Effects of Cannabinoids

The endocannabinoid system is associated with the balance of homeostasis and, as cannabinoids interact with this system, they have been studied, in recent decades, for the treatment of various pathologies (Table 1) ([29,30] (pp. 4–10), (pp. 183–186)). THC has greater bioactivity, although it is also known for the effects of euphoria, relaxation, and changes in sensory and temporal perception. In the case of CBD, and due to the pharmacological effects detailed above, it has been shown to be effective as an anticonvulsant, antipsychotic, anti-inflammatory, antioxidant, neuroprotective, and anxiolytic [4] (p. 5).

These bioactivities lead, among the possible therapeutic applications, those involving neurodegenerative diseases characterized by a loss of neurons, which leads to a decline in motor and cognitive capacity. All this information is also associated with inflammation with a major role in the progression of the disease. Therefore, the properties that CBD exhibits at the level of neurodegeneration, inflammation, and antioxidation may come to be very important in the treatment and stabilization of these diseases, such as, for example, Alzheimer’s, Parkinson and Huntington’s [25] (pp. 8–9).

Another complex pathology that benefits from the bioactivity of these compounds are multiple sclerosis, which is an autoimmune disease characterized by demyelination that occurs in CNS [20] (p. 1143), leading to characteristic symptoms such as spasticity that causes severe pain and difficulty sleeping [31] (p. 18). Cannabinoids are able to alleviate these symptoms through interaction with the CB1 receptor, reducing the massive release of glutamate, which leads to reduced spasticity, producing analgesic effects [32] (p. 10).

The potential adjuvant therapy with cannabinoids, mainly with CBD, is also studied in epilepsy, but the mechanism of action is not yet fully understood [33] (p. 397). However, the regulation of T-type calcium channels, as well as of PPARs by CBD, may be a possible mechanism, since both are associated with seizures [22] (p. 5).

Moreover, in Tourette’s syndrome, which is a chronic neurological disease characterized by the presence of motor and vocal tics, there were improvements with the administration of cannabinoids [31] (p. 22).

Another area that is much discussed is oncology, but it is more associated with supportive therapy, specifically to reduce some chemotherapy side effects. Effectively, there is already validation of efficacy and safety in pain relief, reduction of nausea and vomiting, and appetite stimulation, associated with chemotherapy and palliative care. THC is able to centrally antagonize 5-HT3 receptors, mediating an anti-emetic effect. It also can activate CB1 receptors, suspending the emetic effects triggered by serotonin and dopamine [31] (pp. 19–20). THC has the ability to stimulate appetite through the activation of CB1 receptors located at the level of the hypothalamus and which are responsible for regulating the energy balance ([32,34] (pp. 9–10), (p. 140)).

There is evidence that these compounds mediate antitumor effects by inhibiting cell proliferation, inducing autophagy-mediated apoptosis, and intervening in the migration, invasion, and metastasis of cancer cells. Thus, in the near future, can lead to the development of new drugs based on these cannabinoid structures, for instance, on CBD, which is an antagonist of GPCR 55 in various types of cancer ([17,25,32] (pp. 11–12), (pp. 10–13), (p. 11)).

Another potential therapeutic application, for example for THC, is the treatment of increased pressure in the eyeball caused by glaucoma, which is an optic neuropathic disease, which leads to irreversible loss of vision [28] (p. 1690).

With regard to inflammatory bowel diseases, such as Crohn’s disease and ulcerative colitis, it is known that CBD has the ability to slow down their progression and improve symptoms such as abdominal pain, diarrhea, and anorexia ([31,34] (p. 21), (pp. 2–4)). In these situations, cannabinoids are able to promote an anti-inflammatory action by acting on CB2 receptors and wound healing acting on CB1 receptors [3] (p. 310).

Another pathology very much associated with cannabinoids is schizophrenia. In this disease, changes occur at the level of the endocannabinoid system, increasing the expression of CB1 receptors. Here, CBD may have important antipsychotic potential as opposed to THC which induces psychosis [31] (pp. 22–23).

With regard to sleep disorders, it is known that THC decreases sleep latency time as well as nocturnal awakenings. In turn, CBD in medium to high doses has a sedative effect, showing potential for the treatment of insomnia [35] (pp. 9–10). Since CBD acts at the level of 5-HT1A receptors through which it plays anxiolytic and antidepressant effects, it can also contribute to stabilizing the sleep cycle [33] (p. 397).

Finally, it is known that the endocannabinoid system is associated with pain control [31] (p. 20). Thus, cannabinoids perform their analgesic effects through the activation of CB1 receptors [3] (pp. 309–310).

### 3.4. Autism Spectrum Disorder

Autism is a complex neurodevelopmental pathology [36] (p. 1) that affects 1% of people over the world [9] (p. 6) and is characterized by difficulties in social interaction/communication and restricted or repetitive patterns of behavior. These characteristics are accompanied by an atypical sensory experience that is estimated to occur in approximately 95% of children with ASD and affect every sensory modality [37] (p. 1).

The most common psychopathological comorbidities include Attention Deficit Hyperactivity Disorder (ADHD), Anxiety Disorders, Depressive Disorders, Bipolar Disorder and Related Disorders, Intellectual Development Disorder, Obsessive-Compulsive Disorder, and Related Disorders, Gender Dysphoria and Disorders in the spectrum of schizophrenia. In addition, there are other medical conditions that can be associated with autism such as epilepsy, sensory and sleep disturbances, immune disorders, neuroinflammation, gastrointestinal disorders, fragile X syndrome, Tourette syndrome, tic disorder, and also, tuberous sclerosis [8] (p. 4).

Although ASDs are considered chronic diseases, symptoms can vary over time with age, development, the environment surrounding the patients, and the interventions to which they are subjected [38] (p. 3).

#### 3.4.1. Etiology

In recent decades, the etiology of ASDs has been widely investigated, however, it is still not fully understood. Currently, it is believed that the cause of these pathologies comes from a set of genetic, epigenetic, and environmental factors [38] (p. 1).

One of the various intervening factors thought to be associated with the etiology of autism is the imbalance in the inhibitory and excitatory systems whose signaling pathways, whether gamma-aminobutyric acid (GABA) or glutamate, are altered and, consequently, influence synaptic function ([39,40] (p. 1), (p. 1)). There is also evidence of alterations at the level of the endocannabinoid system, namely, a decrease in the activity of this system, as discussed below [41] (p. 1). Furthermore, in this pathology, there are still changes that can be detected by neuroimaging and that have led to carry out several studies [42] (pp. 1–12).

Based on published research, the “early brain overgrowth theory” emerged, which defends the existence of a pattern of excessive brain volume growth in early childhood [6] (p. 9). Other studies often report changes in regional gray matter volume in different areas of the brain. In addition, an increase in cortical thickness is found in the frontal cortex and a decrease in the temporal areas [42] (pp. 1–12). Furthermore, the results are consistent regarding the presence of a pattern of general brain under-connectivity, together with local over-connectivity in specific regions, namely, in the frontal and occipital regions [6] (p. 9).

With regard to the genetic component, there are hundreds of genes associated with ASDs. However, they result, most often, from the additive effect of common genetic variants and only 25 to 30% of cases of the disease are associated with rare genetic variants, which include copy number variations (CNVs) and single nucleotide polymorphisms (SNPs) [43] (pp. 3–4). Most proteins originated by these genes are involved in synaptic and neuronal homeostasis, actively intervening in development [44] (p. 5).

In addition to the central symptoms of ASDs, there is a varied prevalence, 9–90%, of gastrointestinal problems, which is much higher than that reported in neurotypical patients and also a higher severity and frequency [45] (pp. 1–2). Despite several limitations associated with meta-analyses of studies of microbiome variations worldwide, some conclusions have already been drawn. In the meta-analysis of Iglesias-Vázquez et al. (2020) [45] (pp. 1–17), it is stated that children with ASDs present dysbiosis in relation to certain bacterial groups and, therefore, may be involved in the development and severity of the disorder. Additionally, deregulation of amino acids and alterations in the metabolism of tryptophan (Trp) and serotonin (increase greater than 25%) were found in ASDs.

The presence of these disturbances has also been associated with pre- and perinatal environmental factors. During pregnancy, a woman enters into a framework of immunosuppression and, therefore, the probability of contracting infections becomes greater [46] (p. 2). Exposure to bacterial and viral infections, especially during the first and second trimester of pregnancy, has also been associated with an increase in the risk of developing ASDs [8] (p. 2). Moreover, maternal age above 40 years and paternal age above 50 years can be risk factors for the development of the pathology, as well as a time period of fewer than 24 months between pregnancies [6] (p. 7). Another relevant issue mentioned as a possible environmental cause is some trace elements. One of the studied elements is zinc, whose scarcity during pregnancy is shown to have a relationship with ASDs. This is due to the fact that it intervenes in fetal growth and development by being associated with the regulation of the immune and antioxidant systems and, therefore, its lack can lead to serious alterations in neurodevelopment [46] (pp. 3–4).

Finally, it is noteworthy that the prevalence of ASDs is approximately four times higher in males compared to females, which may be due to several factors. On one hand, sex seems to influence genetic risk and, on the other hand, symptoms in girls tend to be less evident, culminating in a gap in diagnoses [47] (pp. 1–9).

#### 3.4.2. Treatment

The ASDs tend to have a high prevalence and a significant impact on both the patient’s quality of life and the persons around them. However, there is still no effective pharmacological treatment for the central symptoms of the disease [7] (p. 92).

Non-pharmacological therapy essentially includes cognitive, behavioral, and educational therapies that allow for the mitigation of some symptoms of the disease as well as associated comorbidities [6] (pp. 10–13).

The approved pharmacotherapy does not allow the treatment of the pathology itself, acting only on symptoms and behavior associated with it [6] (p. 13). The Food and Drug Administration (FDA) has approved two drugs for the treatment of irritability associated with ASDs, risperidone, and aripiprazole [44] (pp. 188–189). The antipsychotic risperidone works by antagonizing 5-HT2 receptors and dopamine D2 receptors in the brain. [48] (p. 37). Aripiprazole is an atypical antipsychotic of the third generation that works by combining partial agonism of 5-HT1A receptors and dopamine D2 receptors and antagonism of 5-HT2A receptors [49] (p. 51). Both drugs can cause adverse effects such as drowsiness, increased appetite, weight gain, and extrapyramidal effects ([48,49] (p. 1), (p. 1)). The scarcity of drugs, the incidence of adverse effects and the high number of refractory patients [7] (p. 1) lead to the search for other options that improve or replace conventional therapy [9] (p. 3).

### 3.5. Application of the Cannabinoid Therapy in Autism Spectrum Disorders

In recent years, several studies have been published that demonstrate the role of the endocannabinoid system in the pathophysiology of ASDs [23] (pp. 4–5). CB1 receptors are expressed with high density in brain areas that control social functioning, as emotional responses, social interactions and behaviors adapted to the context [50] (pp. 840–843). Since the activity of the endocannabinoid system is reduced in several ASD models, it may be one of the factors responsible for the difficulties in communication and social interaction present in individuals with this pathology ([23,41] (p. 4), (p. 9)). This statement is supported by studies showing that the levels of endocannabinoids, such as AEA and their derivatives, palmitoylethanolamide, and oleylethanolamide, are reduced in autistic patients ([51,52] (p. 5), (p. 7)). Furthermore, it is known that AEA-mediated endocannabinoid signaling modulates social reward and is dependent on oxytocin. Thus, the AEA deficit in patients with ASD may contribute to the changes that occur in social behavior [53] (p. 14084). The administration of CBD has shown therapeutic potential in this area since this phytocannabinoid inhibits the AEA-degrading enzyme, FAAH, increasing plasma levels of this endocannabinoid [54] (p. 29). In addition, CBD interacts with cannabinoid and other non-cannabinoid brain receptors and may exert beneficial effects on the CNS [52] (p. 7).

Individuals diagnosed with ASDs also show alterations in the immune system, such as an increase in antibodies against CNS proteins and maternal proteins, as well as changes at the inflammatory level, with elevated plasma levels of pro-inflammatory cytokines [18] (p. 2). In these patients, there is an upregulation of the expression of CB2 receptors and a decrease in the expression of NAPE-PLD enzyme in immune cells, which shows the influence of the endocannabinoid system on the immune system [55] (p. 7). The change in activity and phenotype of microglia cells, fundamental in the development of the CNS and that play essential functions for its homeostasis, and which is related to CNS diseases including ASDs, can function as a target in the treatment of autism [19] (p. 4). The administration of CBD results in a decrease in the expression of CB2 receptors, as well as an increase in AEA which contributes to mitigating the immunological alterations [54] (p. 33). Besides, CBD has an anti-inflammatory effect by decreasing the levels of pro-inflammatory cytokines and contributing to a protective phenotype of microglia [19] (p. 3).

In addition to acting on the central symptoms underlying ASDs, CBD interacts with several receptors involved in comorbidities associated with autism. Epilepsy is one of the most common comorbidities affecting between 20 to 30% of individuals with ASDs [27] (p. 2). CBD has anticonvulsant properties targeting voltage-dependent calcium and sodium channels, TRPV1s, and PPARs ([22,27] (p. 2), (p. 5)). This phytocannabinoid also interacts with receptors 5 -HT1A through which it can alleviate the symptoms of depressive and anxiety disorders as well as sleep disturbances that are common in patients with ASDs. ADHD is the comorbidity most frequently associated with ASDs diagnosed in 40 to 70% of autistic children [4] (p. 8). CBD is shown to be effective in reducing hyperactivity and impulsivity and also suggests improvement of the attention span in patients with ADHD [56] (p. 7). Moreover, gastrointestinal diseases that appear regularly in patients with ASDs and cannabinoids can improve the symptomatology of these disorders through their anti-inflammatory and immune system modulating properties [57] (p. 16).

#### 3.5.1. Preliminary Clinical Studies

There has been a growing interest in the use of cannabinoids for the treatment of the central symptoms of ASDs, as well as associated comorbidities. This is reflected in the number of published studies on this topic, whose characteristics and results are described in Table 2. The number of participants (patients included) shows great variability among the analyzed studies. For example, two publications report only individual cases associated with ASDs ([58,59] (p. 5), (p. 2)), but a clinical investigation with the largest number of participants (188), which evaluates the safety and efficacy of cannabinoid use in these patients, is also available [60] (p. 2). Concerning the age of the participants, most studies have children as interveners ([58,59,60,61,62] (p. 5), (p. 2), (p. 2), (p. 1285), (p. 3)). As an exception, there are the articles published by Pretzsch et al. (2019) ([39,40,63] (p. 1399), (p. 3), (p. 1142)) that have adults as participants. In addition to these, two of the studies include children and adults ([64,65] (p. 932), (p. 2)).

Regarding the administered therapy, most studies have as treatment extracts of the plant *C. sativa* L. with an amount of CBD higher than THC. One of the most used ratios is 20:1 of CBD:THC ([59,61] (p. 4), (p. 1285)). However, in the article published by Kuester et al. (2017) [64] (p. 933), where a treatment based on the plant extract with a balanced CBD:THC ratio was followed, they also administered an extract with a high content of CBD and another with a high content of THC to some participants. In four more trials, the research groups chose to administer differently. In two of these, only CBD (600 mg) ([39,63] (p. 1399), (p. 1142)) was used, while the third chose isolated administration of cannabidivarin (CBDV) [40] (p. 2), and in the last, dronabinol (3.62 mg/day) [58] (p. 5).

The administration schedules and doses are quite heterogeneous and the study in which there is a smaller amount of cannabinoids contemplates a daily administration of 8 mg of CBD and 0.4 mg of THC [59] (p. 2), while the study with a greater amount of these compounds predicts a maximum daily administration of 600 mg of CBD and 40 mg of THC [65] (p. 2). It is important to note that two of the studies analyzed do not specify the treatment used or the dose administration schedule ([57,66] (pp. 17–18), (pp. 1–6)).

Duration of the treatment and follow-up are also components that vary widely between studies. Most are observational cohort studies and, therefore, the duration of treatment is different between the various participants ([57,60,61,62,65] (p. 18), (p. 4), (p. 1285), (p. 3), (p. 2)). Overall, the duration of treatment varies from administration of a single dose ([39,40,63] (p. 1399), (p. 2), (p. 1142)) to a follow-up of participants treated with cannabinoids for up to 13 months [61] (p. 1285). The follow-up of participants varies both in terms of time and methods used. The most common is a follow-up at the beginning and at the end of the treatment, however, there are studies that carry on a continuous follow-up ([59,61,62,65] (pp. 2–5), (pp. 1285–1287), (p. 4), (p. 2)). The most used methods of follow-up are the questionnaires directed to the caregivers who evaluate participants’ symptoms through various questions and scales.

The results of the studies suggest that treatment with cannabinoids may exert beneficial effects with respect to the central symptoms of ASDs and symptoms associated with comorbidities. Most studies assess the effect of cannabinoids on comorbidities associated with ASDs revealing their therapeutic properties in the treatment of sleep and behavioral disorders, hyperactivity, anxiety, irritability, and aggressiveness. Many of the studies also demonstrate the beneficial effects of cannabinoids against epilepsy, reducing the frequency and intensity of seizures. The evidence generated by these studies regarding epilepsy is combined with other evidence that allowed the approval of the drug Epidiolex^®^ for the treatment of rare epilepsy syndromes such as Lennox-Gastaut syndrome and Dravet syndrome [67] (p. 1).

Likewise, there are a small number of studies that assess the impact of cannabinoids in the treatment of central symptoms of ASDs (communication and social interaction, and stereotyped or repetitive speech and behaviors). The publication by Kuester et al. (2017) [64] (pp. 932–933), which assessed the improvement of those symptoms, concluded that most participants improved in at least one of these. Another study [61] (p. 1286) published in 2018 evaluated that 60 children with autism showed a 47% improvement in communication problems. Moreover, the study by Fleury-Teixeira et al. (2019) [62] (pp. 4–6) that include 15 participants with communication and social interaction disorders, showed improvements equal to or greater than 15% in this symptomatology in 11 of them. The individual case report that evaluated a 12-year-old child using cannabinoids as complementary therapy also revealed improvements in behavioral and communicative symptoms [59] (p. 5). These results are very promising and may open doors to the possibility of cannabinoids use for the treatment of central symptoms of ASDs since there are no drugs approved yet [4] (p. 3).

In many studies, participants took other medications to treat symptoms associated with autism, such as stimulants, antipsychotics, or antidepressants. With the concomitant use of products based on cannabinoids, there was a huge decrease in the use of these drugs and, in some cases, it even resulted in their discontinuation ([60,61,66] (p. 3), (p. 1286), (p. 2)). These are relevant results since, frequently, the drugs used in the treatment symptoms associated with ASDs, such as atypical antipsychotics, cause serious side effects, for instance, drowsiness, blurred vision, tremors, akathisia, dizziness, or anxiety ([48,49] (p. 1), (p. 1)). However, Fleury-Teixeira et al. (2019) [62] (p. 6) found that four of the 18 study participants had negative results with cannabinoid-based treatment and all of them were taking other medications. Thus, it is thought that these negative results may come from unwanted drug-drug interactions between cannabinoids and other drugs used to treat symptoms associated with ASDs. This type of interaction did not occur in any of the other studies. However, they should always be evaluated so that the results will be not biased.

The most common adverse effects were agitation, irritability, drowsiness, and lack or increase in appetite. These effects may, or may not, be due to cannabinoid treatment as many of the participants were also taking other medications, as discussed above. The majority of adverse reactions observed were mild to moderate and transient. In the analysis by Aran et al. (2019) [61] (pp. 1285–1286) three of 60 participants discontinued cannabinoid treatment because two of them developed marked irritability and one girl suffered a psychotic event after increasing the dose of THC to 0.72 mg/kg/day. Given this situation, the cannabinoid treatment was suspended and an antipsychotic was prescribed, with the total disappearance of the adverse effect after nine days. Moreover, in the study by Bar-Lev Schleider et al. (2019) [60] (p. 3), five children discontinued treatment due to the appearance of adverse effects.

Among the analyzed studies, there are 3 control cases that are part of the same investigation, and that aimed to understand the role of some phytocannabinoids, namely CBD and cannabidivarin (CBDV), in the treatment of ASDs. These randomized studies included one of the groups taking the placebo and have a crossover design. All groups have 34 adult male participants, 17 of whom were diagnosed with ASDs and the other 17 belong to the control group. Two of the published articles analyze the effect of CBD and CBDV on the inhibitory and excitatory systems in the brain while the other assesses the effect of CBD on low-frequency activity and functional brain connectivity (FC) ([39,40,63] (p. 1398), (p. 1), (p. 1141)). It is known that both, CBD and CBDV, intervene in the regulation of the glutamatergic excitatory system and the GABAergic inhibitory system, thus being able to modulate these signaling pathways that are altered in individuals with ASDs ([39,40] (pp. 1398–1399), (pp. 1–2)). The results obtained show that CBD is responsible for the increase of glutamate and glutamine in basal ganglia and decreased dorsomedial prefrontal cortex, regardless of the presence of ASDs. Despite this, CBD decreases gamma-aminobutyric acid (GABA) in basal ganglia and dorsomedial prefrontal cortex in participants diagnosed with ASDs but increases this neurotransmitter in neurologically typical individuals [39] (p. 1403). In the case of CBDV, there are no changes in glutamate and glutamine levels in the dorsomedial prefrontal cortex but there are changes in basal ganglia. In ASD individuals, there are low levels of glutamate in basal ganglia and, therefore, CBDV promotes an increase in glutamate. In contrast, neurotypical individuals present high levels of glutamate in the basal ganglia, which leads CBDV to decrease this neurotransmitter. However, regarding GABA levels, there are no changes at the cortical and subcortical levels after administration of CBDV [40] (pp. 6–8). Furthermore, the excitatory and inhibitory systems play a fundamental role in the maintenance of low fractional amplitude of low-frequency fluctuations (fALFF) and FC. Regarding the low-frequency activity, it was found that CBD induces an increase in fALFF in the cerebellar vermis, which is accompanied by an increase in FC at the subcortical level and a decrease at the cortical level. In the case of CBD, it also causes an increase in fALFF in the right fusiform gyrus, more prominently in individuals with ASDs, but it does not change FC in this region. The difference in sensitivity in participants with ASDs may be due to the impairment of the GABAergic system that occurs in this pathology [63] (pp. 1144–1146).

The analyzed studies have some limitations that must be taken into account as they can corrupt results and, therefore, prevent their possible replication and validation. Most studies are observational and without a control group and, consequently, it is not possible to establish causality between cannabinoid therapy and the improvement in ASDs symptoms. The high number of drugs that are administered concomitantly with cannabinoid therapy also contributes to this factor. Besides, the number of participants in the studies is reduced, which makes the sample not representative. As described above the cannabinoid therapy administered also varies greatly, both in terms of concentration and ratio. Finally, there is high heterogeneity in the symptoms that are evaluated as well as in the measures used to assess them. Thus, although cannabinoid therapy proves to be promising in the treatment of central symptoms and those associated with ASDs it is imperative to carry on randomized and placebo-controlled clinical trials that demonstrate the efficacy and safety of these treatments with more accurate protocols.

#### 3.5.2. Clinical Trials

So far, there are nine studies registered on the ClinicalTrials.gov platform related to the use of cannabinoids in ASDs [68]. These are summarized in Table 3.

From the studies registered, only one is complete with published results [69]. This randomized and controlled clinical trial evaluated 150 patients diagnosed with ASDs and behavioral problems, aged between five and 21 years. Two treatments lasting 12 weeks each, separated by four weeks apart. The therapy administered to the participants was an extract of the complete *C. sativa* L. plant dosed in CBD:THC (20:1), a mixture of pure CBD and THC, also in a ratio of 20:1, or placebo (olive oil solution). Outcomes were assessed using various scales. For behavioral problems, two scales were used. The Home Situations Questionnaire—Autism Spectrum Disorder (HSQ-ASD), which consists of a 24-item questionnaire, aimed at caregivers of patients with ASDs and assesses their behavior and the Clinical Global Impression—Improvement (CGI-I) which evaluates improvement of patients and it is composed of seven items. In addition, the Social Responsiveness Scale—second edition (SRS-2) was used to assess the central symptoms of ASDs and the Autism Parenting Stress Index (APSI) which reflects parental stress associated with symptoms and comorbidities from children with the disease ([69,78], (pp. 1–4)). The results showed that there were no significant differences in the HSQ-ASD and APSI scales between patients receiving cannabinoids and placebo. There was a 49% improvement in CGI-I in participants who received cannabinoids from a complete plant extract and 38% in those who received pure cannabinoids versus 21% in those who received the placebo. Furthermore, there was also a significant improvement in the central symptoms of ASDs in patients treated with extracts of the whole plant evaluated by SRS-2. Thus, it was demonstrated, for the first time, in a randomized and controlled clinical trial that cannabinoids can exert a beneficial effect on symptoms associated with ASDs, especially on behavioral problems [78] (pp. 4–9).

Of the remaining studies registered, most are still in the recruitment phase [73,74,75,76,77]. Still, there is an observational study registered on the platform which, although complete, has not yet published results [71].

The clinical trials cited above investigated mostly children diagnosed with ASDs. However, there are research groups that chose to evaluate adults or are restricted to male participants. Another important parameter for the analysis and comparison of results is that there is high heterogeneity in terms of the number of participants, which ranges from one to 160, and the duration of treatment, which varies between six weeks and five years. Thus, although there are already ongoing clinical trials, the results generated are still scarce and it is necessary to do them on a large scale and long term. Further studies should also include more homogeneous samples in terms of age and prescribed drugs, which will allow more robust data for clinical support.

### 3.6. Adverse Effects of Cannabis

Acute adverse effects are dose-dependent and translate into increased cardiovascular activity, tachycardia, and systemic vasodilation. Additionally, in individuals with pre-diagnosed cardiovascular disease, long-term cannabis use increases the risk of myocardial infarction, cardiomyopathy, angina, and cardiorespiratory arrest ([20,32,79] (p. 1143), (pp. 12–13), (p. 736)).

Furthermore, since smoking is the most used method of cannabis consumption, there are several adverse effects associated with the respiratory system. At the acute level, there is an increase in airway resistance and inflammation as well as the destruction of lung tissue. On a chronic level, there is an increased risk of developing respiratory diseases such as chronic bronchitis and pulmonary emphysema. Finally, since smoke contains a high amount of carcinogenic substances, cannabis use is also associated with lung cancer ([32,79] (p. 15), (p. 736)).

With regard to mental health, consumption of this plant is essentially associated with acute adverse effects such as anxiety or panic attacks. However, long-term use in patients with a family history of psychosis or with pre-existing mental pathologies can lead to the development or exacerbation of diseases such as schizophrenia, bipolar disorder, and depression ([20,32] (pp. 1140–1141), (p. 24)). At the cognitive level, consumption of this plant causes changes in sensory and temporal perception, affects short-term memory, and also has an impact on psychomotor function leading to ataxia and catalepsy [32] (p. 13). Long-term consumption leads to the development of dependence, albeit in a low percentage of individuals [79] (p. 739).

The age groups with the highest cannabis consumption include individuals of reproductive age as well as adolescents ([32,80] (p. 15), (p. 182)). This factor is of important relevance since cannabinoids exert anti-androgenic effects and can suppress sexual maturation [32] (pp. 15–16). It is important to highlight that cannabinoids easily cross the placenta and are secreted through breast milk, hence maternal exposure to cannabis is associated with a reduction in weight gain in pregnant women, as well as in low-birth-weight new-borns [32] (pp. 16–17).

Briefly, the medicinal use of cannabinoids is essentially associated with adverse effects such as drowsiness, tiredness, dry mouth, blurred vision, anxiety, and cognitive effects [32] (p. 12). However, most of the adverse effects mentioned above are due to THC and, therefore, it is possible to avoid them through the use of low amounts of this and subsequent titration, as well as through the use of CBD that is able to suppress these deleterious effects [32] (p. 7). A summary overview is given in Table 4.

### 3.7. Approved Drugs

Currently, several drugs, whose active ingredients are cannabinoids, are marketed worldwide. Some of these compounds are synthetic, such as nabilone and dronabinol, and others are natural substances extracted from the plant *C. sativa* L.

Nabilone is a synthetic cannabinoid similar to THC and is used as an active ingredient in Cesamet^®^ and Canemes^®^ [81] (p. 8). Cesamet^®^ was approved in 1992 by the FDA [81] (p. 21) for the treatment of chemotherapy-induced nausea and vomiting in patients who do not respond to conventional antiemetics. In the European Union (EU) it is only available under an *Exception Use Permit* ([81,82] (p. 9), (p.5)).

Dronabinol is a synthetic form of THC [81] (p. 8) used to treat anorexia associated with weight loss in patients with Acquired Immune Deficiency Syndrome (AIDS) and also to treat chemotherapy-induced nausea and vomiting in patients who do not respond to conventional antiemetics [81] (p. 9). This compound is present as an active ingredient in two drugs. Marinol^®^, which was approved in 1985 by FDA [81] (p. 21), comes in the form of capsules for oral administration ([81,83] (p. 9), (p. 1)) while Syndros^®^ was only approved in 2016 and is an oral solution with 5 mg/mL of dronabinol. ([81,84] (p. 9), (p. 1)). In the EU it is also only available by an *Exception Use Permit* [81].

There are two more drugs approved that contain cannabinoids derived from the *C. sativa* L. plant, Epidiolex^®^ and Sativex^®^. Epidiolex^®^ was approved in 2018 by FDA [81] (p. 21) and is an oral solution of CBD used in the treatment of seizures associated with Lennox-Gastaut syndrome and Dravet syndrome, in patients aged two years and over ([67,81] (p. 1), (p. 9)). In turn, Sativex^®^ is formulated as an oral spray solution based on a standardized extract of THC and CBD, in a ratio of 2.7 mg and 2.5 mg, respectively. This drug is used to improve spasticity-related symptoms in multiple sclerosis patients’ refractory to first-line drugs ([81,85] (p. 9), -). While Epidiolex^®^ was only approved as an orphan drug by the European Medicines Agency (EMA) in 2019, Sativex^®^ is already in use in several European countries since 2010 through the mutual recognition procedure [81].

## 4. Discussion

Considering that ASDs are a set of neurodevelopmental diseases with a high prevalence that can severely affect the quality of life of patients and those around them, it is essential to find strategies for the treatment of various symptoms associated either with the disease itself or with the comorbidities that accompany it. Until now, the approved drugs still have several limitations already described which has generated a great demand for other options or complements to conventional therapy. Among them, the possibility of using parts of the *C. sativa* L. or its extracts, as well as its phytocannabinoids isolated or in mixtures with different ratios, have been investigated, often more with the aim of supporting the already protocoled therapy, particularly for changes in the endocannabinoid system. Regarding this last topic, there are two aspects for which scientific evidence has been found which could bring a forthright benefit. One is associated with the fact that the serum levels of endocannabinoids when they are decreased can contribute to impairing communication and social interaction, and the other involves the upregulation of CB2 receptors and the downregulation of the expression of the NAPE-PLD enzyme in immune cells, which may contribute to immunological changes common in ASDs. Thus, in this context, since CBD can inhibit the FAAH enzyme, increasing the levels of the endocannabinoid AEA and decreasing the expression of CB2 receptors, it results in a benefit that will certainly become an important way to reduce some of the symptoms. In addition, this cannabinoid has anti-inflammatory properties and, therefore, lowers levels of pro-inflammatory cytokines, and also contributes to a protective microglial phenotype.

In regard to this possibility being also applied at the level of the central symptoms of this pathology, there are already some studies that indicate cannabinoids as having a relevant potential in the development of new therapeutic forms. The evidence generated is still considered narrow since there are several limitations associated with these studies, namely, the heterogeneity in the study design, the number of participants, the concentration, components and dose of the treatment administered, and also the symptoms evaluated and the tools used to evaluate them. Thus, it is essential to perform clinical trials which develop results that contribute to generating evidence concerning the efficacy and safety of cannabinoid use in ASDs. There are some ongoing clinical trials, but published results are still scarce. Furthermore, there is a high variability of the participants’ characteristics. It is expected that there will be several clinical trials in the process of publishing results in early 2022 and although they still have a low number of participants, they may bring relevant information.

To our knowledge, this review provides, for the first time, data and discussion regarding the correlation of ASDs and cannabinoids from basic science studies to human translational research. A clearer understanding of these issues may have a revolutionary impact on new therapeutics, the target being the improvement of everyday life of people in the spectrum, for example, by supporting some core behavioral changes. Nevertheless, the mentioned gaps should be filled with more data, corrections, and recommendations that properly contribute to and advance the work of previous studies.

Therefore, it becomes a key priority to carry on future large-scale and long-term clinical trials that include more homogeneous samples and methodologies, which bring robust data into the clinic and health decision-making.

## Figures and Tables

**Table 1:** Summary of the therapeutic potential of cannabinoids in different diseases and symptoms.

Diseases and Symptoms	Therapeutic Potential of Cannabinoids
Alzheimer’s Disease	Anti-inflammatory Neuroprotector Antioxidant
Parkinson’s Disease	
Huntington’s Disease	
Multiple Sclerosis	Antispastic Analgesic
Epilepsy	Anticonvulsant
Tourette’s Syndrome	Improvement of symptomatology
Cancer	Analgesic Antiemetic Appetite stimulator Antitumor
Glaucoma	Intraocular pression reduction
Inflammatory Bowel Diseases	Anti-inflammatory Healing
Schizophrenia	Antipsychotic
Sleep Disorders	Decrease sleep latency and nocturnal awakenings Sedative
Pain	Analgesic
Post-Traumatic Stress Disorder	Anxiolytic
Nausea and Vomiting	Antiemetic
Anorexia	Appetite stimulator

**Table 2:** Characteristics and results of preliminary studies with extracts of cannabis, cannabinoids, and their derivatives.

Title	Publication Year	Type of Study	Number of Participants	Average Age	Administrated Treatment	Daily Dose	Duration of Treatment and Follow-Up	Outcomes (% of Decrease of Symptoms)	Adverse Effects (Number of Participants)	Reference
Use of dronabinol (delta-9-THC) in autism: A prospective single-case-study with an early infantile autistic child	2010	Individual Case Study	1	6 years old	Dronabinol dissolved in sesame oil	Initial: 1 drop in the morning (0.62 mg) Final: 1 drop in the morning, 1 drop in the middle of the day, and 3 drops in the evening (3.62 mg)	Treatment: 6 months Follow-up: initial and after 6 months	Irritability: 55.5% Lethargy: 26.7% Stereotyped behaviour: 33.3% Hyperactivity: 56.2% Inappropriate speech: 28.6%	NR	[58]
Oral cannabis extracts as a promising treatment for the core symptoms of autism spectrum disorder: Preliminary experience in Chilean patients	2017	Case Series Study	21	9 years old and 10 months	15 participants: extract with CBD:THC (1:1) 4 participants: extract with high CBD content 2 participants: extract with high THC content	NR	Treatment: 3 to 12 months Follow-up: after 6, 7 months, on average	CGI-I e APSI: 66.7% Central symptoms of ASDs: >50%, in at least one of the symptoms Sensory disturbances, food acceptance, sleep disturbances, and seizures: >50%	Agitation: 2 Irritability: 1	[64]
Brief Report: Cannabidiol-Rich Cannabis in Children with Autism Spectrum Disorder and Severe Behavioral Problems—A Retrospective Feasibility Study	2018	Retrospective Cohort Study	60	11 years old and 8 months	Plant extract with CBD:THC (20:1), dissolved in olive oil	2 administrations per day (1.8 ± 1.6 mg/kg/day of CBD and 0.22 ± 0.14 mg/kg/day of THC) 3 administrations per day (3.8 ± 2.6 mg/kg/day of CBD and 0.29 ± 0.22 mg/kg/day of THC)	Treatment: 7 to 13 months Follow-up: continuous	Behaviour: 61% Anxiety: 39% Communication: 47% HSQ-ASD: 29% APSI: 33%	Hypervigilance: 8 Agitation: 5 Irritability: 5 Decrease appetite: 5	[61]
Oral Cannabidiol Use in Children with Autism Spectrum Disorder to Treat Related Symptoms and Co-morbidities	2019	Prospective Cohort Study	53	11 years old	Oil with 30% of cannabinoids, CBD:THC (1:20)	16 mg/kg of CBD (maximum of 600 mg daily) 0.8 mg/kg of THC (maximum of 40 mg daily)	Treatment: 66 days, on average Follow-up: biweekly telephone interviews	Self-injury and rage attacks: 67.6% Hyperactivity: 68.4% Sleep disorders: 71.4% Anxiety: 47.1% Overall improvement: 74.5%	Somnolence: 12 Decrease appetite: 6 Appetite increase: 4	[65]
Real-life experience of medical cannabis treatment in autism: analysis of safety and efficacy	2019	Prospective Cohort Study	188	12 years old and 9 months	66 participants: Oil with 30% of CBD and 1.5% of THC 46 participants with insomnias: Oil with 3% of THC	66 participants: 3 administrations per day (79.5 ± 6.5 mg of CBD and 4.0 ± 3.0 mg of THC, per dose) 46 participants with insomnia: 1 administration at evening (5.0 ± 4.5 mg of THC, per dose)	Treatment: 6 months Follow-up: after 1 and 6 months	Quality of life: 35.5% Mood: 21.5% Ability to perform activities of daily living: 16.5% Sleep quality: 21.4% Concentration: 14% Agitation: 91% Rage attacks: 90.3% Seizures: 15.4% (total improvement: 84.6%)	Agitation: 6 Somnolence: 3 Psychoactive effects: 3 Appetite increase: 3 Digestion problems: 3 Dry mouth: 2 Decrease appetite: 2	[60]
Effects of CBD-Enriched Cannabis sativa Extract on Autism Spectrum Disorder Symptoms: An Observational Study of 18 Participants Undergoing Compassionate Use	2019	Prospective Cohort Study	18	10 years old and 9 months	Plant extract dosed in CBD:THC (75:1), administered as oral capsules	2 administrations per day (4,55 mg/kg/day of CBD and 0,06 mg/kg/day of THC, on average)	Treatment: 6 to 9 months Follow-up: initial and monthly	ADHD: 86.7% Behavioural disorders: 73.3% Motor deficits: 83.3% Autonomy deficits: 66.7% Communication and social interaction deficits: 73.3% Cognitive deficits: 86.7% Sleep disorders: 100% Seizures: 60% (total improvement: 40%)	Somnolence: 3 Irritability: 3 Diarrhea: 1 Appetite increase: 1 Conjunctival hyperemia: 1 Increased body temperature: 1 Nocturia: 2	[62]
Medical Cannabis in the Treatment of Patients with Autism Spectrum Disorder	2020	Case Series Study	20	NR	Medicinal cannabis	NR	NR	Improvement in: All points of ACGIC Epilepsy Pain Sleep Mood Aggressiveness Concentration	Unspecified adverse effects: 3	[66]
A pediatric patient with autism spectrum disorder using cannabinoid extracts as complementary therapy: a case report	2020	Individual Case Study	1	15 years old	CBE dosed in CBD:THC (20:1), dissolved in olive oil	Administração de 0.2 mL duas vezes ao dia (4 mg de CBD e 0.2 mg de THC, por dose)	Follow-up: contínuo de 6 meses	Improvement in: Quality of life Behavioral and communicative symptoms Anxiety Sleep disorders Weight gain	NR	[59]
Autism Spectrum Disorder and Medical Cannabis: Review and Clinical Experience	2020	Retrospective Cohort Study	32	NR	Medicinal or Hemp-based Cannabis Products	NR	NR	Epilepsy: 91% Aggressiveness: 60%	Worsening of obsessive-compulsive and repetitive behavior, insomnia or mania: 4	[57]
Effects of cannabidiol on brain excitation and inhibition systems; a randomized placebo-controlled single-dose trial during magnetic resonance spectroscopy in adults with and without autism spectrum disorder.	2019	Case- Control Study	34, of which: 17 with ASD 17 without ASD (control)	31 years old and 3 months	Liquid oral dose of CBD or Placebo	600 mg of CBD or Placebo	Treatment: 2 doses separated by, at least, 13 days Follow-up: at the time of administration and after 2 h	CBD increases glutamate in the basal ganglia and decreases in the prefrontal cortex in participants with ASD and control CBD decreases GABA in basal ganglia and prefrontal cortex in ASD subjects and increases in control subjects	NR	[39]
Effects of cannabidivarin (CBDV) on brain excitation and inhibition systems in adults with and without Autism Spectrum Disorder (ASD): a single dose trial during magnetic resonance spectroscopy	2019	Case- Control Study	34, of which:17 with ASD 17 without ASD(control)	31 years old and 3 months	Liquid oral dose of CBDV or Placebo	600 mg of CBDV or Placebo	Treatment: 2 doses separated by, at least, 13 days Follow-up: at the time of administration and after 2 h	CBDV with no impact on GABA and glutamate levels in the prefrontal cortex CBDV alters basal ganglia glutamate levels negatively correlated with baseline glutamate levels	NR	[40]
The effect of cannabidiol (CBD) on low-frequency activity and functional connectivity in the brain of adults with and without autism spectrum disorder (ASD)	2019	Case- Control Study	34, of which: 17 with ASD 17 without ASD (control)	31 years old and 3 months	Liquid oral dose of CBD or Placebo	600 mg of CBD or Placebo	Treatment: 2 doses separated by, at least, 13 days Follow-up: at the time of administration and after 2 h	CBD prominently increases fALFF in the cerebellar vermis and right fusiform gyrus in subjects with ASD CBD increases FC in the cerebellar vermis but not in the right fusiform gyrus	NR	[63]

Legend: APSI—Change in Autism Parenting Stress Index; CBD—Cannabidiol; CGI-I—Clinical Global Impression-Improvement; HSQ-ASD—Home Situations Questionnaire-Autism Spectrum Disorder; NR—Not Reported; ASD—Autism Spectrum Disorder; THC—∆9-tetrahidrocannabinol. ACGIC—Autism Caregiver Global Impression of Change; ADHD—Attention-deficit/hyperactivity disorder; CBDV—Cannabidivarin; GABA—Gamma-aminobutyric Acid; FC—Functional Connectivity; fALFF—Fractional Amplitude of Low-frequency Fluctuations.

**Table 3:** Clinical trials characteristics.

Title	Identifier	Type of Study	State	Study Population	Number of Participants	Duration	Expected Completion Date	Results	Reference
Cannabinoids for Behavioural Problems in Autism Spectrum Disorder: A Double-Blind, Randomized, Placebo-controlled Trial with Crossover	NCT02956226	Randomized Interventional Study (Clinical Trial) Quadruple mask Parallel	Complete	Patients with ASD and behavioral problems, between 5 and 21 years old	150	3 months	27 December 2018	Published	[69]
Shifting Brain Excitation-Inhibition Balance Through the Endocannabinoid System in Men with Autism Spectrum Disorder (ASD) and in Healthy Controls	NCT03537950	Randomized Interventional Study (Clinical Trial) Double mask Crossover	Unknown	Patients with ASD, between 18 and 50 years old, male	38	5 to 6 months	27 April 2019	Not Published	[70]
Medical Cannabis Registry and Pharmacology	NCT03699527	Observational Study (Prospective Cohort Study)	Complete	Patients with ASD under 21 years old	119	5 years	15 January 2020	Not Published	[71]
Safety and Tolerability of GWP42006 in Children and Young Adults with Autism Spectrum Disorder	NCT03849456	Interventional Study (Clinical trial) Open Label	Terminated	Patients with ASD, between 4 and 18 years old	1	52 weeks	26 May 2020	Not Published	[72]
Cannabidivarin (CBDV) vs. Placebo in Children with Autism Spectrum Disorder (ASD)	NCT03202303	Randomized Interventional Study (Clinical Trial) Parallel Double mask	In recruitment	Patients with ASD, between 5 and 18 years old	100	12 weeks	30 September 2021	Not Published	[73]
A Double-Blind, Crossover Trial of Cannabidiol to Treat Severe Behavior Problems in Children with Autism	NCT04517799	Randomized Interventional Study (Clinical Trial) Quadruple mask Crossover	In recruitment	Patients with ASD and severe behavioral problems, between 7 and 14 years old, male	30	16 weeks	31 December 2021	Not Published	[74]
A Phase 2 Study of Cannabidiol as a New Treatment for Autism Spectrum Disorder in Children and Adolescents	NCT03900923	Interventional Study (Clinical trial) Open Label	In recruitment	Patients with ASD, between 7 and 17 years old	30	6 weeks	1 July 2022	Not Published	[75]
An Exploratory, Phase 2, Randomized, Double-blind, Placebo-controlled Trial to Investigate the Safety and Efficacy of Cannabidiol Oral Solution (GWP42003 P; CBD-OS) in Children and Adolescents with Autism Spectrum Disorder	NCT04745026	Randomized Interventional Study (Clinical Trial) Quadruple mask Parallel	In recruitment	Patients with ASD, between 6 and 17 years old	160	12 weeks	30 March 2023	Not Published	[76]
Cannabidiol Study in Children with Autism Spectrum Disorder (CASCADE): A Double-Blind, Placebo-Controlled Study to Investigate Efficacy and Safety of Cannabidiol in Children and Adolescents With Autism	NCT04520685	Randomized Interventional Study (Clinical Trial) Triple mask Crossover	In recruitment	Patients with ASD, between 5 and 17 years old	70	27 weeks	1 June 2023	Not Published	[77]

Legend: ASD—Autism Spectrum Disorder.

**Table 4:** Adverse effects of cannabis.

Affected Area	Adverse Effects
Cardiovascular System	Increase cardiovascular activity Tachycardia Systemic vasodilation Increased risk of myocardial infarction, cardiomyopathy, angina, and cardiorespiratory arrest
Respiratory System	Increased inflammation and airway resistance Destruction of lung tissue Increased risk of developing chronic bronchitis and pulmonary emphysema Increased risk of developing lung cancer
Mental Health	Anxiety and panic attacks Exacerbation or development of schizophrenia, bipolar disorder, and depression in vulnerable patients
Cognition	Change in sensory and temporal perception Short-term memory alteration Change in psychomotor function Dependency
Hormonal System, Fertility and Maternity	Anti-androgenic effects Reduction of pregnant weight gain Low-birth-weight new-borns

## Abbreviations

2-AG	2-Arachidonylglycerol;
5-HT	Serotonin;
AA	Arachidonic Acid;
AC	Adenylate Cyclase;
ADHD	Attention Deficit Hyperactivity Disorder;
AEA	*N*-Arachidonoylethanolamide;
APSI	Autism Parenting Stress Index;
ASD	Autism Spectrum Disorder;
ATP	Adenosine Triphosphate;
cAMP	Cyclic Adenosine Monophosphate;
CBD	Cannabidiol;
CBDV	Cannabidivarin;
CGI-I	Clinical Global Impression—Improvement;
CNP	Peripheral Nervous System;
CNS	Central Nervous System;
CNVs	Copy Number Variations;
CVS	Cardiovascular System;
DAG	Diacylglycerol;
DAGL	Diacylglycerol Lipase;
EU	European Union;
FAAH	Fatty Acid Amide Hydrolase;
fALFF	Fractional Amplitude of Low-Frequency Fluctuations;
FC	Functional Brain Connectivity;
FDA	Food and Drug Administration;
GABA	Gamma-Aminobutyric Acid;
GPCRs	Orphan G Protein-Coupled Receptors;
HSQ-ASD	Home Situations Questionnaire—Autism Spectrum Disorder;
MAGL	Monoacylglycerol Lipase;
MAPK	Mitogen-Activated Protein Kinase;
NAPE	*N*-Acyl-Phosphatidylethanolamine;
NAPE-PLD	*N*-Acyl-Phosphatidylethanolamine-Specific Phospholipase D;
PKA	Protein Kinase A;
PPARγs	Nuclear Receptors Activated by Peroxisome Proliferators Type γ;
Receptor CB1	Cannabinoid Receptor Type 1;
Receptor CB2	Cannabinoid Receptor Type 2;
SNPs	Single Nucleotide Polymorphisms;
SRS-2	Social Responsiveness Scale—Second Edition;
THC	∆9-Tetrahydrocannabinol;
Trp	Tryptophan;
TRPV1s	Transient Receptors Potential Cation Channel Subfamily V Member 1.

## References

[1] Klumpers L.E., Thacker D.L. (2019). A brief background on cannabis: From plant to medical indications. J. AOAC Int..

[2] Pollio A. (2016). The Name of Cannabis: A Short Guide for Nonbotanists. Cannabis Cannabinoid Res..

[3] Bonini S.A., Premoli M., Tambaro S., Kumar A., Maccarinelli G., Memo M., Mastinu A. (2018). Cannabis sativa: A comprehensive ethnopharmacological review of a medicinal plant with a long history. J. Ethnopharmacol..

[4] Poleg S., Golubchik P., Offen D., Weizman A. (2018). Cannabidiol as a suggested candidate for treatment of autism spectrum disorder. Prog. Neuro-Psychopharmacol. Biol. Psychiatry.

[5] American Psychiatric Association (2013). Diagnostic and Statistical Manual of Mental Disorders.

[6] Lord C., Elsabbagh M., Baird G., Veenstra-Vanderweele J. (2018). Autism spectrum disorder. Lancet.

[7] Young N., Findling R.L. (2015). An update on pharmacotherapy for autism spectrum disorder in children and adolescents. Curr. Opin. Psychiatry.

[8] Sharma S.R., Gonda X., Tarazi F.I. (2018). Autism Spectrum Disorder: Classification, diagnosis and therapy. Pharmacol. Ther..

[9] Loss C.M., Teodoro L., Rodrigues G.D., Moreira L.R., Peres F.F., Zuardi A.W., Crippa J.A., Hallak J.E.C., Abílio V.C. (2021). Is Cannabidiol during Neurodevelopment a Promising Therapy for Schizophrenia and Autism Spectrum Disorders?. Front. Pharmacol..

[10] Bundesinstitut für Arzneimittel und Medizinprodukte (2018). German Pharmacopoeia, Cannabis, Flor–Monograph. Cannabisblüten Cannabis Flos.

[11] Mcpartland J.M. (2018). Cannabis Systematics at the Levels of Family, Genus, and Species. Cannabis Cannabinoid Res..

[12] Kinghorn A.D., Falk H., Gibbons S., Kobayashi J. (2017). 103-Phytocannabinoids-Unraveling the Complex Chemistry and Pharmacology of *Cannabis sativa*. Progress in the Chemistry of Organic Natural Products.

[13] Gould J. (2015). The Cannabis Crop. Nature.

[14] European Medicines Agency, Herbal Medicinal Products Committee (2006). Guideline on Good Agricultural and Collection Practice (GACP) for Starting Materials of Herbal Origin. https://www.ema.europa.eu/en/good-agricultural-collection-practice-starting-materials-herbal-origin.

[15] Maroon J., Bost J. (2018). Review of the neurological benefits of phytocannabinoids. Surg. Neurol. Int..

[16] Lu D., Potter D.E. (2017). Cannabinoids and the Cannabinoid Receptors: An Overview. Handbook of Cannabis and Related Pathologies: Biology, Pharmacology, Diagnosis, and Treatment.

[17] Zou S., Kumar U. (2018). Cannabinoid receptors and the endocannabinoid system: Signaling and function in the central nervous system. Int. J. Mol. Sci..

[18] Brigida A.L., Schultz S., Cascone M., Antonucci N., Siniscalco D. (2017). Endocannabinod signal dysregulation in autism spectrum disorders: A correlation link between inflammatory state and Neuro-Immune alterations. Int. J. Mol. Sci..

[19] Araujo D.J., Tjoa K., Saijo K. (2019). The Endocannabinoid System as a Window into Microglial Biology and Its Relationship to Autism. Front. Cell. Neurosci..

[20] Cohen K., Weizman A., Weinstein A. (2019). Positive and Negative Effects of Cannabis and Cannabinoids on Health. Clin. Pharmacol. Ther..

[21] Loprinzi P.D., Zou L., Li H. (2019). The endocannabinoid system as a potential mechanism through which exercise influences episodic memory function. Brain Sci..

[22] Chonhofen P., Bristot I.J., Crippa J.A., Hallak J.E.C., Zuardi A.W., Parsons R.B., Klamt F. (2018). Cannabinoid-Based Therapies and Brain Development: Potential Harmful Effect of Early Modulation of the Endocannabinoid System. CNS Drugs.

[23] Aran A., Cayam-Rand D. (2020). Medical cannabis in children. Rambam Maimonides Med. J..

[24] Appendino G. (2020). The early history of cannabinoid research. Rend. Lincei.

[25] Pisanti S., Malfitno M., Ciaglia E., Ranieri R., Cuomo G., Abate M., Faggiana G., Proto M.C., Fiore D., Laezza C. (2017). Cannabidiol: State of the art and new challenges for therapeutic applications. Pharmacol. Ther..

[26] Devinsky O., Cilio M.R., Cross H., Fernandez-Ruiz J., French J., Hill C., Katz R., Marzo V.D., Jutras-Aswad D., Notcutt W.G. (2014). Cannabidiol: Pharmacology and potential therapeutic role in epilepsy and other neuropsychiatric disorders. Epilepsia.

[27] Gu B. (2017). Cannabidiol provides viable treatment opportunity for multiple neurological pathologies of autism spectrum disorder. Glob. Drugs Ther..

[28] Fraguas-Sánchez A.I., Torres-Suárez A.I. (2018). Medical Use of Cannabinoids. Drugs.

[29] White C.M. (2019). A Review of Human Studies Assessing Cannabidiol’s (CBD) Therapeutic Actions and Potential. J. Clin. Pharmacol..

[30] Bridgeman M.B., Abazia D.T. (2017). Medicinal Cannabis: History, Pharmacology, and Implications for the Acute Care Setting. Pharm. Ther..

[31] Stasiłowicz A., Tomala A., Podolak I., Cielecka-Piontek J. (2021). Cannabis sativa L. As a natural drug meeting the criteria of a multitarget approach to treatment. Int. J. Mol. Sci..

[32] Breijyeh Z., Jubeh B., Bufo S.A., Karaman R., Scrano L. (2021). Cannabis: A Toxin-Producing Plant with Potential Therapeutic Uses. Toxins.

[33] Oberbarnscheidt T., Miller N.S. (2020). The Impact of Cannabidiol on Psychiatric and Medical Conditions. J. Clin. Med. Res..

[34] Goyal H., Singla U., Gupta U., May E. (2017). Role of cannabis in digestive disorders. Eur. J. Gastroenterol. Hepatol..

[35] Babson K.A., Sottile J., Morabito D. (2017). Cannabis, Cannabinoids, and Sleep: A Review of the Literature. Curr. Psychiatry Rep..

[36] Robertson C.E., Baron-Cohen S. (2017). Sensory perception in autism. Nat. Rev. Neurosci..

[37] Chung S., Son J.-W. (2020). Visual Perception in Autism Spectrum Disorder: A Review of Neuroimaging Studies. J. Korean Acad. Child Adolesc. Psychiatry.

[38] Mukherjee S.B. (2017). Autism Spectrum Disorders—Diagnosis and Management. Indian J. Pediatrics.

[39] Pretzsch C.M., Freyberg J., Voinescu B., Lythgoe D., Horder J., Mendez M.A., Wichers R., Ajram L., Ivin G., Heasman M. (2019). Effects of cannabidiol on brain excitation and inhibition systems; a randomised placebo-controlled single dose trial during magnetic resonance spectroscopy in adults with and without autism spectrum disorder. Neuropsychopharmacology.

[40] Pretzsch C.M., Freyberg J., Voinescu B., Lythgoe D., Horder J., Mendez M.A., Wichers R., Ajram L., Ivin G., Heasman M. (2019). Effects of cannabidivarin (CBDV) on brain excitation and inhibition systems in adults with and without Autism Spectrum Disorder (ASD): A single dose trial during magnetic resonance spectroscopy. Transl. Psychiatry.

[41] Zou M., Liu Y., Xie S., Wang L., Li D., Li L., Wang F., Zhang Y., Xia W., Sun C. (2021). Alterations of the endocannabinoid system and its therapeutic potential in autism spectrum disorder. Open Biol..

[42] Lukito S., Norman L., Carlisi C., Radua J., Hart H., Simonoff E., Rubia K. (2020). Comparative meta-analyses of brain structural and functional abnormalities during cognitive control in attention-deficit/hyperactivity disorder and autism spectrum disorder. Psychol. Med..

[43] Eapen V., Nicholls L., Spagnol V., Mathew N.E. (2017). Current status of biological treatment options in Autism Spectrum Disorder. Asian J. Psychiatry.

[44] Masi A., Demayo M.M., Glozier N., Guastella A.J. (2017). An Overview of Autism Spectrum Disorder, Heterogeneity and Treatment Options. Neurosci. Bull..

[45] Iglesias-Vázquez L., van Ginkel Riba G., Arija V., Canals J. (2020). Composition of Gut Microbiota in Children with Autism Spectrum Disorder: A Systematic Review and Meta-Analysis. Nutrients.

[46] Yoon S.H., Choi J., Lee W.J., Do J.T. (2020). Genetic and Epigenetic Etiology Underlying Autism Spectrum Disorder. J. Clin. Med..

[47] Loomes R., Hull L., Mandy W.P.L. (2017). What Is the Male-to-Female Ratio in Autism Spectrum Disorder? A Systematic Review and Meta-Analysis. J. Am. Acad. Child Adolesc. Psychiatry.

[48] RISPERDAL (risperidone)-Prescribing Information. https://www.accessdata.fda.gov/drugsatfda_docs/label/2009/020272s056,020588s044,021346s033,021444s03lbl.pdf.

[49] ABILIFY (aripiprazol)-Prescribing Information. https://www.accessdata.fda.gov/drugsatfda_docs/label/2014/021436s038,021713s030,021729s022,021866s023lbl.pdf.

[50] Chakrabarti B., Persico A., Battista N., Maccarrone M. (2015). Endocannabinoid Signaling in Autism. Neurotherapeutics.

[51] Karhson D.S., Krasinska K.M., Dallaire J.A., Libove R.A., Phillips J.M., Chien A.S., Garner J.P., Hardan A.Y., Parker K.J. (2018). Plasma anandamide concentrations are lower in children with autism spectrum disorder. Mol. Autism.

[52] Aran A., Eylon M., Harel M., Polianski L., Nemirovski A., Tepper S., Schnapp A., Cassuto H., Wattad N., Tam J. (2019). Lower circulating endocannabinoid levels in children with autism spectrum disorder. Mol. Autism.

[53] Wei D., Lee D.Y., Cox C.D., Karsten C.A., Penagarikano O., Geschwind D.H., Gall C.M., Piomelli D. (2015). Endocannabinoid signaling mediates oxytocin-driven social reward. Proc. Natl. Acad. Sci. USA.

[54] Schultz S., Siniscalco D. (2019). Endocannabinoid system involvement in autism spectrum disorder: An overview with potential therapeutic applications. AIMS Mol. Sci..

[55] Siniscalco D., Sapone A., Giordano C., Cirillo A., de Magistris L., Rossi F., Fasano A., Bradstreet J.J., Maione S., Antonucci N. (2013). Cannabinoid receptor type 2, but not type 1, is up-regulated in peripheral blood mononuclear cells of children affected by autistic disorders. J. Autism Dev. Disord..

[56] Cooper R.E., Williams E., Seegobin S., Tye C., Kuntsi J., Asherson P. (2017). Cannabinoids in attention-deficit/hyperactivity disorder: A randomised-controlled trial. Eur. Neuropsychopharmacol..

[57] Mostafavi M., Gaitanis J. (2020). Autism Spectrum Disorder and Medical Cannabis: Review and Clinical Experience. Semin. Pediatric Neurol..

[58] Kurz R., Blaas K. (2010). Use of dronabinol (delta-9-THC) in autism: A prospective single-case-study with an early infantile autistic child. Cannabinoids.

[59] Ponton J.A., Smyth K., Soumbasis E., Llanos S.A., Lewis M., Meerholz W.A., Tanguay R.L. (2020). A pediatric patient with autism spectrum disorder and epilepsy using cannabinoid extracts as complementary therapy: A case report. J. Med. Case Rep..

[60] Bar-Lev Schleider L., Mechoulam R., Saban N., Meiri G., Novack V. (2019). Real life Experience of Medical Cannabis Treatment in Autism: Analysis of Safety and Efficacy. Sci. Rep..

[61] Aran A., Cassuto H., Lubotzky A., Wattad N., Hazan E. (2019). Brief Report: Cannabidiol-Rich Cannabis in Children with Autism Spectrum Disorder and Severe Behavioral Problems—A Retrospective Feasibility Study. J. Autism Dev. Disord..

[62] Fleury-Teixeira P., Caixeta F.V., Da Silva L.C.R., Brasil-Neto J.P., Malcher-Lopes R. (2018). Effects of cbd-enriched cannabis sativa extract on autism spectrum disorder symptoms: An observational study of 18 participants undergoing compassionate use. Front. Neurol..

[63] Pretzsch C.M., Freyberg J., Voinescu B., Lythgoe D., Horder J., Mendez M.A., Wichers R., Ajram L., Ivin G., Heasman M. (2019). The effect of cannabidiol (CBD) on low-frequency activity and functional connectivity in the brain of adults with and without autism spectrum disorder (ASD). J. Psychopharmacol..

[64] Kuester G., Vergara K., Ahumada A., Gazmuri A.M. (2017). Oral cannabis extracts as a promising treatment for the core symptoms of autism spectrum disorder: Preliminary experience in Chilean patients. J. Neurol. Sci..

[65] Barchel D., Stolar O., De-Haan T., Ziv-Baran T., Saban N., Fuchs D.O., Koren G., Berkovitch M. (2019). Oral cannabidiol use in children with autism spectrum disorder to treat related symptoms and Co-morbidities. Front. Pharmacol..

[66] Mcvige J., Headd V., Alwaidy M., Lis D., Kaur D., Albert B., Mechtler L. (2020). Medical cannabis in the treatment of patients with autism spectrum disorder. Neurology.

[67] EPIDIOLEX (cannabidiol) Oral Solution-Prescribing Information. https://www.accessdata.fda.gov/drugsatfda_docs/label/2018/210365lbl.pdf.

[68] Home-ClinicalTrials.gov. https://clinicaltrials.gov/.

[69] Cannabinoids for Behavioral Problems in Children with ASD (NCT02956226). NCT02956226.

[70] Shifting Brain Excitation-Inhibition Balance in Autism Spectrum Disorder (NCT03537950). NCT03537950.

[71] Medical Cannabis Registry and Pharmacology (NCT03699527). NCT03699527.

[72] Safety and Tolerability of Cannabidivarin (CBDV) in Children and Young Adults with Autism Spectrum Disorder (NCT03849456). NCT03849456.

[73] Cannabidivarin (CBDV), vs. Placebo in Children with Autism Spectrum Disorder (ASD) (NCT03202303). NCT03202303.

[74] Trial of Cannabidiol to Treat Severe Behavior Problems in Children with Autism (NCT04517799). NCT04517799.

[75] Cannabidiol for ASD Open Trial (NCT03900923). NCT03900923.

[76] Trial to Investigate the Safety and Efficacy of Cannabidiol Oral Solution (GWP42003-P; CBD-OS) in Children and Adolescents with Autism Spectrum Disorder (NCT04745026). NCT04745026.

[77] CASCADE: Cannabidiol Study in Children with Autism Spectrum Disorder (NCT04520685). NCT04520685.

[78] Aran A., Harel M., Cassuto H., Polyansky L., Schnapp A., Wattad N., Shmueli D., Golan D., Castellanos F.X. (2021). Cannabinoid treatment for autism: A proof-of-concept randomized trial. Mol. Autism.

[79] Sachs J., Mcglade E., Yurgelun-Todd D. (2015). Safety and Toxicology of Cannabinoids. Neurotherapeutics.

[80] Campbell C.T., Phillips M.S., Manasco K. (2017). Cannabinoids in Pediatrics. J. Pediatric Pharmacol. Ther..

[81] Medical Use of Cannabis and Cannabinoids: Questions and Answers for Policymaking. http://www.emcdda.europa.eu/system/files/publications/10171/20185584_TD0618186ENN_PDF.pdf.

[82] CESAMET (nabilone) Capsules, for Oral Administration-Charateristics. https://www.accessdata.fda.gov/drugsatfda_docs/label/2006/018677s011lbl.pdf.

[83] MARINOL (dronabinol) Capsules, for Oral Use-Prescribing Information. https://www.accessdata.fda.gov/drugsatfda_docs/label/2017/018651s029lbl.pdf.

[84] SYNDROS (dronabinol) Oral Solution-Prescribing Information. https://www.accessdata.fda.gov/drugsatfda_docs/label/2017/205525s003lbl.pdf.

[85] Sativex Oralmucosal Spray. https://www.medicines.org.uk/emc/product/602/smpc#gref.

